# Imported Arbovirus Infections in Spain, 2009–2018 

**DOI:** 10.3201/eid2604.190443

**Published:** 2020-04

**Authors:** Francesca F. Norman, César Henríquez-Camacho, Marta Díaz-Menendez, Sandra Chamorro, Diana Pou, Israel Molina, Josune Goikoetxea, Azucena Rodríguez-Guardado, Eva Calabuig, Clara Crespillo, Inés Oliveira, José-Antonio Pérez-Molina, Rogelio López-Velez, for the Redivi Study Group

**Affiliations:** Ramón y Cajal University Hospital, Madrid, Spain (F.F. Norman, C. Henríquez-Camacho, S. Chamorro, J.-A. Pérez-Molina, R. López-Velez);; La Paz-Carlos III Hospital, Madrid (M. Díaz-Menendez, C. Crespillo); Vall d’Hebron-Drassanes, Barcelona, Spain (D. Pou, I. Molina, I. Oliveira);; Cruces University Hospital, Bilbao, Spain (J. Goikoetxea);; Asturias Central University Hospital, Oviedo, Spain (A. Rodríguez-Guardado);; La Fe de Valencia University Hospital, Valencia, Spain (E. Calabuig)

**Keywords:** dengue, chikungunya, Zika, travel, immigration, viruses, Spain, arboviruses, vector-borne infections

## Abstract

To determine the epidemiologic and clinical characteristics of patients in Spain with imported arbovirus infections, we analyzed 22,655 records from a collaborative network for January 2009–December 2018. Among 861 arbovirus infections, 845 were monoinfections (456 [53%] dengue, 280 [32.5%] chikungunya, 109 [12.7%] Zika) and 16 (1.8%) were co-infections. Most patients were travelers (56.3%) or immigrants returning to Spain after visiting friends or relatives (31.3%). Median patient age was 37 years; most (62.3%) were women and some (28.6%) had received pretravel advice. Only 12 patients were immunosuppressed. Six cases (all dengue monoinfections, none in immunosuppressed patients) were severe. Since 2014, nondengue arbovirus infections increased; until 2016, chikungunya and Zika were most common. Imported arbovirus infections (mostly dengue) were frequently diagnosed, although increased chikungunya and Zika virus infections coincided with their introduction and spread in the Americas. A large proportion of cases occurred in women of childbearing age, some despite receipt of pretravel advice.

In recent decades, pathogenic flaviviruses (yellow fever virus, dengue [DENV], and Zika [ZIKV]) and the alphavirus chikungunya (CHIKV) have become the most common agents of emerging arbovirus diseases among humans. Their geographic distribution, epidemiologic patterns, and modes of transmission overlap considerably, and because of increased human travel and migration, outbreaks have been reported in non–arbovirus-endemic areas. Proposed drivers of virus epidemics include factors that may affect vector distribution, such as climate change, wars/social change, and poverty (decreased vector control measures) (*1*). Although some factors are unlikely to occur in non–arbovirus-endemic settings, such as Europe, emergence of these infectious diseases in these regions may result from viremic persons with imported infections and presence of competent *Aedes albopictus* mosquitoes (*2*). In nontropical areas, the potential international threat of these viruses is increased by the possibility of nonvectorborne transmission, such as transfusional, sexual, and vertical transmission (with possible severe fetal damage) (*3*). Travelers are at high risk for infection by vectorborne diseases and may contribute to the spread of imported diseases in non–disease-endemic areas (*4*).

Our objective with this study was to describe the epidemiologic and clinical characteristics of patients in Spain with a confirmed diagnosis of imported arbovirus infection. For data, we used the Red Cooperativa para el Estudio de las Infecciones Importadas por Viajeros e Inmigrantes (+Redivi) network (http://www.redivi.es), a specialized network for imported infectious diseases in Spain.

## Methods

We based our analysis on a cohort included in the +Redivi network. The network initially comprised 4 centers; however, new centers have been included over the years. Currently, +Redivi comprises 22 health centers (1 primary care center and 21 hospitals, specialized and not specialized in travel medicine) in 8 regions of Spain that share a common online database in which new cases of imported infections are registered. A unique data collection sheet is used to gather information about patient demographics, trip characteristics (destination, return date, duration, type of traveler), receipt of pretravel advice, receipt of antimalaria antiprophylaxis (when applicable), reason for consultation, and final diagnosis variables. Because patients were deidentified at the time of study inclusion, we could not link data back to patients. Cases recorded were those in which the final diagnosis was considered to be an imported disease associated with travel (for symptomatic and asymptomatic patients). Returning travelers/immigrants could account for >1 case in the database if they had traveled >1 time and received a new diagnosis of an imported disease. +Redivi classified persons attending a first consultation as follows: immigrant (person living in Spain but born in any other country), VFR (visiting friends and relatives) immigrant (immigrant traveling back from his/her country of birth after visiting friends and relatives), VFR traveler (Spanish traveler returning from his/her first-degree relative’s country of birth), and travelers (conventional international tourists returning from travel, expatriates, and missionaries).

We included in our analysis cases recorded in +Redivi during January 2009–December 2018. We identified patients with a diagnosis of arbovirus infection and compared 2 main populations: those with imported arbovirus infection and those with nonarbovirus infection. Acute/recent arbovirus infection was diagnosed for patients with exposure risk and a virus-positive PCR, positive IgM, evidence of seroconversion, or nonstructural (NS) 1 antigen detection for DENV. A diagnosis of ZIKV infection based on positive IgM required a finding of negative DENV IgM and vice versa or confirmation with a neutralization assay (not available at all centers). We classified cases as severe if there was evidence of shock and plasma leakage, severe hemorrhage, or organ impairment (according to the World Health Organization definition) (*5*). We performed a descriptive analysis to assess the distribution of these groups according to patient sex, age, type of case (immigrant, VFR, or traveler), time from arrival in Spain to medical consultation, immunosuppression, country of birth, duration of travel, date of first arrival in Spain, length of travel, date of return from travel, travel destination, and rate of pretravel advice receipt.

We expressed qualitative data as relative and absolute frequencies and quantitative data as median and interquartile ranges with 95% CIs. To compare categorical variables, we used χ^2^ and Fischer exact tests when appropriate; to compare continuous variables, we used the Student *t* test (for data that were normally distributed) or the Mann-Whitney U test (for data that were not normally distributed). We used bivariate analyses to compare demographic characteristics and performed multivariable logistic regression to determine the association of exposures and outcomes. We obtained adjusted odds ratios (aORs) and 95% CIs and set the threshold for statistical significance as a 2-sided p<0.05.

## Results

We analyzed 22,655 records from the +Redivi database; patients included 12,460 (55.0%) immigrants, 3,627 (16.0%) VFR immigrants, 414 (1.8%) VFR travelers, and 6,154 (27.2%) travelers. The most prevalent diagnoses in +Redivi were Chagas disease (18.7%), eosinophilia (5.4%), *Plasmodium falciparum* malaria (4.9%), latent tuberculosis (4.4%), schistosomiasis (4.1%), strongyloidiasis (3.9%), and arbovirus infections (3.8%). Among travelers, the second most prevalent diagnosis was arbovirus infections (8.2%), which followed acute nonspecific diarrhea (12.3%).

For a total of 861 (3.8%) cases (16 co-infections and 845 monoinfections), the final diagnosis was acute/recent arbovirus infection. Patients with monoinfections included 23 (2.7%) immigrants, 308 (36.5%) VFR immigrants, 21 (2.5%) VFR travelers, and 493 (58.3%) travelers. Arbovirus infections were mainly acquired in South America, Central America, and the Caribbean (p<0.001), except for travelers, who acquired most of their arbovirus infections (mostly dengue) in Southeast Asia (Tables 1, 2).

**Table 1:** Main characteristics of patients with arbovirus monoinfections included in study of arbovirus infections, Spain, 2009–2018*

Characteristic	Immigrants, n = 23	VFR immigrants, n = 308	VFR travelers, n = 21	Travelers, n = 493	p value
Median age (IQR), y	25 (14-48)	40 (34-48)	29 (11-37)	35 (29-43)	<0.001
Sex, no. (%)					<0.001
F	13(56.5)	240 (77.9)	10 (47.6)	263 (53.4)	
M	10 (43.5)	68 (22.1)	11 (52.4)	230 (46.7)	
Etiologic virus, no.					<0.001
Dengue	14	93	9	340	
Chikungunya	8	162	7	103	
Zika	1	53	5	50	
Region, no. (%)†					<0.001
Sub-Saharan Africa	4 (17.4)	12 (3.9)	2 (9.5)	42 (8.5)	
North America	0	0	0	2 (0.4)	
Central America and Caribbean	5 (21.7)	94 (30.5)	9 (42.9)	128 (26.0)	
South America	10 (43.5)	192 (62.3)	6 (28.6)	84 (17.1)	
South-central Asia	3 (13.0)	6 (2.0)	0	61 (12.4)	
Eastern Asia	0	0	0	2 (0.4)	
Southeast Asia	1 (4.4)	4 (1.3)	3 (14.3)	166 (33.7)	
Australasia	0	0	1 (4.8)	4 (0.8)	
Europe	0	0	0	2 (0.4)	
Median travel days, no. (IQR)	0	30 (27–55)	30 (21–75)	23 (15–50)	<0.001
Median weeks until first consultation, no. (IQR)	1.2 (0.6–4.3)	2 (0.9–5.8)	2.6 (1.3–4.4)	1.1 (0.4–3.6)	<0.001
Travel >30 d, no. (%)	0	135 (44.3)	10 (47.6)	164 (33.3)	0,.05
Pretravel advice, no. (%)	0	21 (6.9)	3 (14.3)	218 (44.3)	<0.001
Immunosuppression, no. (%)	1 (4.4)	4 (1.3)	2 (9.5)	5 (1.0)	0.008

*IQR, interquartile range; VFR, visiting friends and relatives. †Data from Red Cooperativa para el Estudio de las Infecciones Importadas por Viajeros e Inmigrantes (+Redivi, http://www.redivi.es).

**Table 2:** Main characteristics of patients with arbovirus monoinfections, by virus type, Spain, 2009–2018*

Characteristic	Dengue, n = 456	Chikungunya, n = 280	Zika, n = 109	p value
Median age (IQR), y	34 (29-43)	41 (34-52)	36 (30-43)	<0.001
Sex, no. (%)				0.001
F	262 (57.5)	198 (70.7)	66 (60.6)	
M	194 (42.5)	82 (29.3)	43 (39.4)	
Type of patient				<0.001
Immigrant	14	8	1	
VFR immigrant	93	162	53	
VFR traveler	9	7	5	
Traveler	340	103	50	
Region, no. (%)				<0.001
Sub-Saharan Africa	39 (8.6)	16 (5.7)	5 (4.6)	
North America	1 (0.2)	0	1 (0.9)	
Central America and Caribbean	89 (19.6)	94 (33.6)	53 (48.6)	
South America	105 (23.1)	139 (49.6)	48 (44.0)	
South-central Asia	58 (12.8)	11 (3.9)	1 (0.9)	
Eastern Asia	2 (0.4)	0	0	
Southeast Asia	156 (34.3)	17 (6.1)	1 (1.0)	
Australasia	3 (0.7)	2 (0.7)	0	
Europe	1 (0.2)	1 (0.4)	0	
Median no. travel days (IQR)	25 (15-36)	31 (25-66)	30 (20-59)	<0.001
Median no. weeks until first consultation (IQR)	1.1 (0.4-3.1)	3 (1.0-7.1)	1.2 (0.6-3.3)	<0.001
Travel >30 d, no. (%)	133 (30.1)	135 (50.2)	41 (38.0)	<0.001
Pretravel advice, no. (%)	175 (39.7)	55 (20.4)	12 (11.1)	<0.001
Immunosuppression (%)	6 (1.3)	4 (1.4)	2 (1.8)	0.919

*IQR, interquartile range; VFR, visiting friends and relatives.

Regarding reason for medical consultation after travel, most patients with arbovirus infections reported fever, followed by myalgia. Six cases, all caused by DENV, were severe; no fatal cases were recorded. A small proportion of asymptomatic patients (53/845, 6.3%) sought a health examination and received a diagnosis of recent arbovirus infection.

Patients with arbovirus monoinfection were a median 37 years of age (interquartile range 30–46 years), and the proportion of women (62.2%; p<0.001) was higher than that of men. Of 526 women, 415 (78.9%) were in the age range to be fertile (15–50 years of age). Among patients with arbovirus monoinfection, only 12 (1.4%) were immunosuppressed (6 because of HIV infection, 3 because of medication, 3 because of other unspecified reason, and none because of transplant). Data on duration of travel and time elapsed from arrival in Spain to consultation are provided (Tables 1, 2). Regarding pretravel advice, 242 (28.6%) patients with an arboviral monoinfection had received advice.

The risk for arbovirus infection was not related to immunosuppression or duration of travel (Table 3). The risk was higher among female (aOR 1.40; 95% CI 1.21–1.61) than male patients and lower among patients who had received pretravel advice than among those who had not (aOR 0.65; 95% CI 0.55–0.77).

**Table 3:** Analysis of variables potentially associated with arbovirus infection (dengue, Zika, and chikungunya virus), Spain, 2009–2018*

Variable	Univariate analysis			Multivariate analysis	
	OR (95% CI)	p value		aOR (95% CI)†	p value
Type of patient					
Immigrant	Referent	NA		Referent	NA
VFR immigrant	50.18 (32.80–76.78)	<0.001		47.15 (30.78–72.22)	<0.001
VFR traveler	28.89 (15.86–52.65)	<0.001		33.53 (18.20–61.77)	<0.001
Traveler	47.09 (30.96–71.62)	<0.001		45.18 (29.68–68.77)	<0.001
Age, y	1.01 (1.01–1.02)	<0.001		1.01 (1.00–1.01)	0.003
Female sex	1.46 (1.27–1.69)	<0.001		1.40 (1.21–1.61)	<0.001
Immunosuppression, yes/no‡	0.28 (0.16–0.49)	<0.001		0.61 (0.34–1.10)	0.099
Pretravel advice, yes/no‡§	0.68 (0.58–0.80)	<0.001		0.65 (0.55–0.77)	<0.001
Travel, d§	1.00 (0.99–1.00)	0.807		0.99 (0.99–1.00)	0.900

*aOR, adjusted odds ratio; NA, not applicable; OR, odds ratio; VFR, visiting friends and relatives. †For type of patient, age, female sex, and immunosuppression. ‡Ratios reflect risk for those who answered yes compared with those who answered no. §For travelers and VFR only.

After 2014, cases of non-DENV arbovirus infection reported in +Redivi increased; CHIKV and ZIKV were the most common arbovirus infections until 2016 (Figure). During the peak of the ZIKV outbreak (mainly December 2015–November 2016), there were 226 cases of arbovirus infections: 85 (37.6%) ZIKV, 82 (36.3%) DENV, 51 (22.6%) CHIKV, and 8 (3.5%) co-infections.

**Figure:**
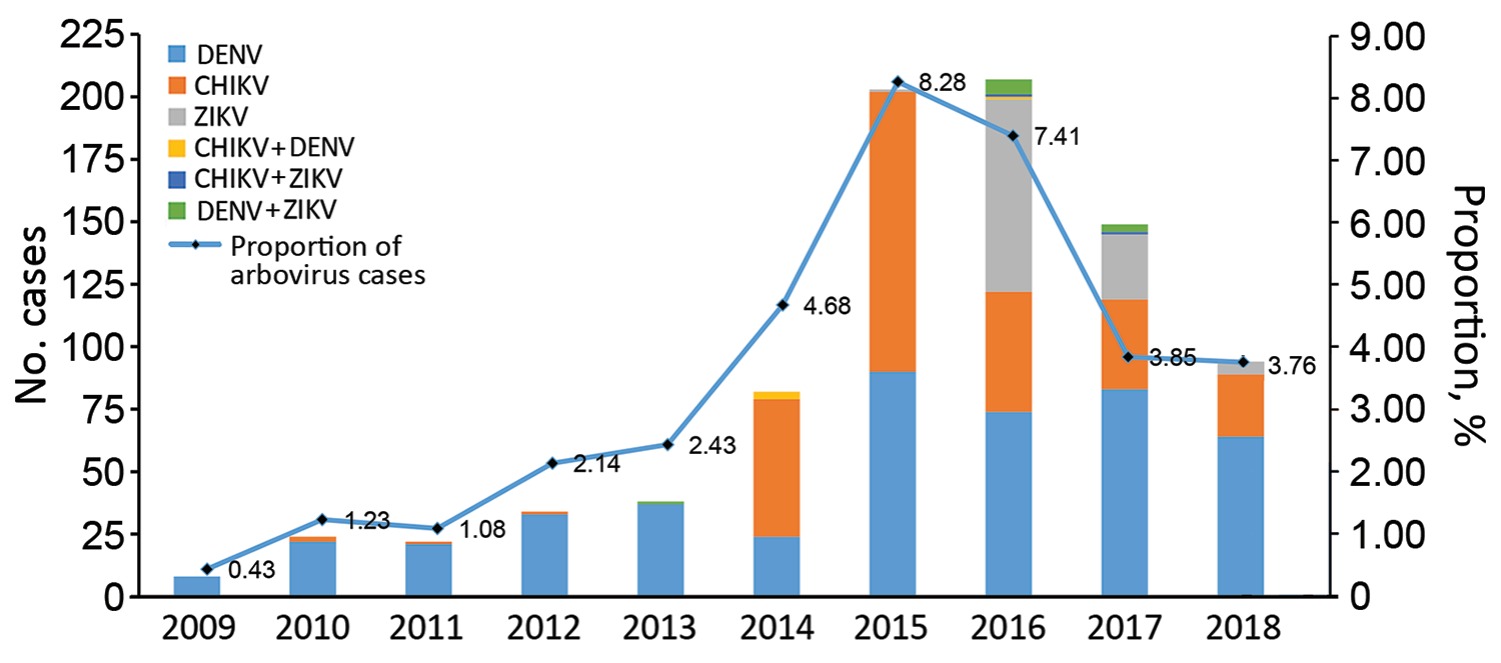
Proportion of arbovirus monoinfections and co-infections, by virus type and year, compared with total number of infections registered in the Red Cooperativa para el Estudio de las Infecciones Importadas por Viajeros e Inmigrantes (+Redivi, http://www.redivi.es) network, Spain, 2009–2018. A) Numbers of arbovirus infections; B) proportion of arbovirus infections compared with total number of infections registered.

### Dengue

A total of 456 (53%) DENV monoinfections were recorded; the proportion of infected women (262 women, 57.5%, p = 0.009) was higher than that of men. Of 262 women, 222 (84.7%) were 15–50 years of age, and of these, only 85 (38.3%) had received pretravel advice. Among patients with dengue infection, 3 had a previous diagnosis of HIV infection, 2 were immunosuppressed secondary to medication, and 1 was immunosuppressed for unspecified cause. The 6 patients with severe nonfatal dengue acquired the infection in South America (66.7%) and Southeast Asia (33.3%); none were immunosuppressed.

### Chikungunya

A total of 280 (32.5%) CHIKV monoinfections were recorded; the proportion of women (198 women, 70.7%; p<0.001) was higher than that of men. Of 198 women, 136 (68.7%) were 15–50 years of age, and of these, only 23 (16.9%) had received pretravel advice. Among patients with CHIKV infection, 2 had a previous diagnosis of HIV infection and 2 were immunosuppressed (unspecified cause).

### Zika

A total of 109 (12.7%) ZIKV monoinfections were recorded; the proportion of women (66 women, 60.6%; p = 0.128) was higher than that of men. Of 66 women, 57 (86.4%) were 15–50 years of age, and of these, only 7 (12.3%) had received pretravel advice. No cases in pregnant women were recorded. Among patients with ZIKV infection, 1 had a previous diagnosis of HIV infection and 1 was immunosuppressed secondary to medication.

### Co-infections

At least 16 (1.8%) patients were co-infected with >1 arbovirus (some probable co-infections with positive serology results for DENV and ZIKV were also registered, but these cases could not be confirmed). DENV and ZIKV co-infection occurred in 10 patients and DENV and CHIKV co-infection in 4. Two patients were co-infected with CHIKV and ZIKV. Most patients were travelers (9/16, 56.3%) or VFR immigrants (5/16, 31.3%). The 10 DENV/ZIKV co-infections were acquired in South America (3), Central America and the Caribbean (3), Australasia (1), Southeast Asia (1), sub-Saharan Africa (1), and south-central Asia (1). The 4 DENV/CHIKV co-infections were acquired in South America (3) and Central America and the Caribbean (1). The 2 CHIKV/ZIKV co-infections were acquired in south-central Asia and South America. No co-infections in immunosuppressed patients were recorded.

## Discussion

In recent years, imported emerging arbovirus infections have become a concern because of the increasing number of cases and the possibility of introduction and local transmission in non–arbovirus-endemic areas; several outbreaks have been documented in non–arbovirus-endemic areas, including several countries in Europe (*6*,*7*). We report data on ≈850 infections caused by arboviruses over a 10-year period (January 2009 to December 2018) registered at centers belonging to a specialized network for imported infectious diseases in Spain. Imported arbovirus infections were among the top 10 established diagnoses in this national network (despite the large proportion of immigrants included in the network, for whom acute arbovirus infection is an infrequent diagnosis); among travelers, arbovirus infection was the second most frequent diagnosis.

Most cases occurred in conventional travelers, followed by VFRs; the small proportion of cases in immigrants may reflect recent acute (nonviremic) infections. Among VFR immigrants, infection with DENV was relatively less frequent than infection with other arboviruses; whereas, for all other groups, the reverse was true. This distribution may reflect a hypothetical protected state if these patients had already experienced multiple DENV infections before immigration. On the other hand, some of the acute infections acquired by persons in this group may have resulted from waning or incomplete immunity to DENV because homotypic reinfections, as well as poor in vitro neutralizing activity of immune human serum for different strains within a single serotype, have been reported (*8*,*9*). These issues could be further explored by investigating the arboviral serostatus of these patients before travel. 

As expected, most cases overall were caused by DENV, although in recent years, and coinciding with the introduction of CHIKV and ZIKV in the Western Hemisphere, increased infections caused by CHIKV and ZIKV have been registered. In our study, CHIKV accounted for most of the arbovirus infections diagnosed during 2015, and ZIKV caused most of the arbovirus infections diagnosed during 2016, mirroring the epidemiologic situation and public concern at the time (ZIKV infection could account for some of the asymptomatic patients who may have sought care for reasons such as preconception or predelivery testing on return from travel) (*10*). Although the network’s catchment area had increased over the years, the proportion of arbovirus infections compared with the total was considered, and increased arbovirus infections were observed during 2015–2017; a downward trend in 2018 reflected global epidemics and increased awareness of these infections.

We found an increased risk for arbovirus infection among women, possibly reflecting increased diagnosis of symptomatic infections. Previous studies have also described increased frequency of arbovirus infections in women compared with men, and female sex has been identified as a key risk factor associated with severe arthralgia caused by CHIKV infection (*11*–*13*). However, these findings are not consistent; other studies describing complicated infections have found that among patients with CHIKV infection admitted to an intensive care unit, the greater proportion were male (*14*). These issues, as well as the presence of other possible contributing factors such as underlying conditions, should be explored further. 

As expected, having received pretravel information was a protective factor; however, a high proportion of patients did not seek pretravel advice, an area in need of improvement and identified in other studies (*15*). Although no vaccines are readily available for use in travelers, simple measures may limit the burden of some of these arbovirus infections, such as avoidance of mosquito bites and the correct diagnosis and education of patients who may transmit the infection to others (e.g., sexual transmission of ZIKV). Of note, many cases occurred in women of childbearing age (in some instances despite pretravel advice), an issue of concern given the association of ZIKV and possibly DENV infection with increased risk for congenital malformations (*16*,*17*). Although we found no records of ZIKV infections in pregnant women, cases of congenital ZIKV infection have been reported in Spain (*18*).

Available data about the outcome of arbovirus disease in immunosuppressed patients is limited. In our study, data were registered for 12 immunosuppressed patients with arbovirus disease; we found no association between infection and immune status, and none of the severe cases (all dengue) occurred in immunosuppressed patients. According to published data, CHIKV in solid organ transplant recipients seems to have a benign course and favorable outcome (*19*,*20*), whereas data for DENV in these patients are less clear. Case descriptions report uncomplicated outcomes, whereas a recent literature review reported a significantly higher incidence of severe dengue and a higher mortality rate among transplant patients with dengue (*21*–*23*). A recent report of DENV, ZIKV, and CHIKV infections diagnosed in hematopoietic stem cell transplant recipients and oncohematologic patients found prolonged viremia in those with DENV and viruria in those with ZIKV; the most common complication was thrombocytopenia, but all patients survived without sequelae (*24*). However, other case reports reveal less favorable outcomes and even atypical presentations: a fatal pseudotumoral form of ZIKV meningoencephalitis in a heart transplant recipient has been reported (*25*). 

With respect to arbovirus infection in HIV-positive patients, data are scarce, although favorable outcomes have been reported (*20*,*26*,*27*). In a study of 43 HIV-positive patients with confirmed ZIKV infection, no hospitalizations, complications, or deaths were found, and CD4 cell counts and HIV viral load did not differ after ZIKV infection (*28*). Regarding arbovirus infections in patients immunosuppressed for other reasons, available data are extremely limited and no firm conclusions can be drawn.

Of interest, we found 16 patients co-infected with multiple arboviruses, reflecting co-circulation and transmission by the same species of DENV, ZIKV, and CHIKV vector during the main outbreak periods in Latin America and the Caribbean. No cases were severe. During periods of intense transmission, co-infections are not infrequent or unexpected (*29*). A recent study that used reverse transcription PCR to investigate patients with acute undifferentiated febrile illness during the CHIKV outbreak in Haiti found that ≈10% of the children with CHIKV infection during the study period were infected with another arbovirus (ZIKV, DENV, or Mayaro virus) (*30*). These dual infections were diagnosed only after culture of the samples, and the authors concluded that that could suggest a low viral load of the co-infecting virus. In addition, the possibility of sequential infections acquired in areas of co-circulation has been previously noted, and sequential infections in patients returning from travel have been described (*31*). Further studies would be needed to determine whether co-infections, sequential infections, or both may be associated with a less favorable outcome.

Some study limitations include the inability to identify which case-patients were viremic at diagnosis. The database included confirmed diagnoses, but diagnosis of some cases may have been based on detection of virus IgM (with or without IgG; i.e., recent acute infections), and so the proportion of viremic patients who may be considered to potentially pose a risk for ongoing transmission in areas of Spain where competent vectors are present was not known. Because only imported cases were registered, possible secondary cases resulting from sexual transmission were not included, probably leading to underestimation of the real extent of imported disease. In some cases, cross-reactions between antibodies to the flaviviruses DENV and ZIKV may have posed a diagnostic challenge (neutralization assays were available only at some centers), and some cases of probable co-infections were excluded and considered monoinfections. Also, previous yellow fever vaccination was not recorded for most patients and was therefore not analyzed, although prior vaccination would be expected to yield possible false-positive IgG results and should not have interfered in the classification of cases as acute or recent infections (which was based on IgM, seroconversion, PCR, or NS1 antigen for DENV). Recent yellow fever vaccination may yield false-positive IgM results for DENV and ZIKV, but most of these infections in this series were acquired in regions where such vaccination was not required (Asia, Caribbean, Australasia). Basic information on main presenting symptoms was recorded, but more detailed information on individual symptoms, enabling a possible comparison with regard to presentation of each arbovirus infection, was not possible.

Despite study limitations, we present some aspects of a large series of imported arbovirus infections diagnosed at specialized centers in Spain. Those imported infections may pose a risk for further transmission in areas where competent *Ae. albopictus* mosquitoes are established, as illustrated by the recent diagnosis of autochthonous DENV and CHIKV infections in several southern European countries such as France, Italy, Croatia, and Spain (*2*,*32*). In 2018, the first 6 cases of locally acquired dengue were reported in Spain, 5 in patients who had traveled to Cádiz and Murcia in southern Spain and 1 in a patient from Barcelona (*2*,*33*). In 2019, an additional case of autochthonous DENV was diagnosed in Catalonia (*34*). The virus was probably introduced into these areas by viremic travelers. In past decades, *Ae. albopictus* mosquito populations have increased in areas of the European Union and are active in several provinces in mainland Spain. *Ae. aegypti* mosquitoes*,* the main vector for DENV transmission, have been introduced in areas of Europe, are present in Madeira (Portugal), and have been identified in the Canary Islands (Spain), although after control measures were implemented locally in 2017, no further detections in the Canary Islands were reported (*2*). Recently, probable sexual transmission of DENV in an area without documented presence of mosquito vectors was reported in Madrid, Spain (*35*).

These cases highlight the value of having a high index of suspicion and the need for rapid diagnosis of arbovirus infections, which may be aided in specific settings (e.g., emergency departments) by the availability of rapid diagnostic tests to detect specific antigens such as the NS1 DENV antigen. Early detection of imported infections in viremic patients could enhance the study of virus genomics through virus sequencing and phylogenetics, which may be used to investigate infectious disease outbreaks and contribute to transmission chain tracking. Viremic patients could also be instructed to protect themselves from insect bites to prevent further transmission in areas where competent vectors are established. 

The number of ZIKV infections in women of childbearing age, despite these women having received pretravel advice, also poses a significant public health concern, coupled with the possible risk for sexual transmission from a partner arriving from a risk area, even several months after travel (*36*). Although sexual transmission of DENV seems to be a rare route of transmission and the risk is considered extremely low, clinicians should consider this possibility for patients with DENV and no compatible travel history and should advise patients with DENV that the risk for transmission via this route may be minimized through safe sex practices. 

These data highlight the challenges posed by these imported infections and the need for improvements, such as the establishment of specific protocols for screening potential blood and organ donors and the enhancement of laboratory capacities. The strengthening of information sources (community-based information and online resources) should be prioritized, not only for at-risk travelers, such as women of fertile age and immunosuppressed patients, but also for physicians in non–arbovirus-endemic areas faced with specific diagnostic and management challenges.
